# Revelation of genetic diversity and structure of wild *Elymus excelsus* (Poaceae: Triticeae) collection from western China by SSR markers

**DOI:** 10.7717/peerj.8038

**Published:** 2019-11-12

**Authors:** Yanli Xiong, Wenhui Liu, Yi Xiong, Qingqing Yu, Xiao Ma, Xiong Lei, Xinquan Zhang, Daxu Li

**Affiliations:** 1 College of Animal science and Technology, Sichuan Agricultural University, Chengdu, China; 2 Qinghai Academy of Animal Science and Veterinary Medicine, Key Laboratory of Superior Forage Germplasm in the Qinghai-Tibetan Plateau, Xi-ning, China; 3 Sichuan Academy of Grassland Sciences, Chengdu, China

**Keywords:** *Elymus excelsus*, SSR, Geographical groups, Genetic diversity, Population structure, Environmental adaptation

## Abstract

Hosting unique and important plant germplasms, the Qinghai-Tibet Plateau (QTP), as the third pole of the world, and Xinjiang, located in the centre of the Eurasian continent, are major distribution areas of perennial Triticeae grasses, especially the widespread *Elymus* species. *Elymus excelsus* Turcz. ex Griseb, a perennial forage grass with strong tolerance to environmental stresses, such as drought, cold and soil impoverishment, can be appropriately used for grassland establishment due to its high seed production. To provide basic information for collection, breeding strategies and utilization of *E. excelsus* germplasm, microsatellite markers (SSR) were employed in the present study to determine the genetic variation and population structure of 25 wild accessions of *E. excelsus* from Xinjiang (XJC) and the QTP, including Sichuan (SCC) and Gansu (GSC) of western China. Based on the 159 polymorphic bands amplified by 35 primer pairs developed from three related species, the average values of the polymorphic information content (PIC), marker index (MI), resolving power (Rp), Nei’s genetic diversity (H) and Shannon’s diversity index (I) of each pair of primers were 0.289, 1.348, 1.897, 0.301 and 0.459, respectively, validating that these SSR markers can also be used for the evaluation of genetic diversity of *E. excelsus* germplasms, and demonstrating the superior versatility of EST-SSR vs. G-SSR. We found a relatively moderate differentiation (*F*_*st*_ = 0.151) among the XJC, SCC and GSC geo-groups, and it is worth noting that, the intra-group genetic diversity of the SCC group (*H*_*e*_ = 0.197) was greater than that of the GSC (*H*_*e*_ = 0.176) and XJC (*H*_*e*_ = 0.148) groups. Both the Unweighted Pair Group Method with Arithmetic (UPGMA) clustering and principal coordinates analysis (PCoA) divided the 25 accessions into three groups, whereas the Bayesian STRUCTURE analysis suggested that *E. excelsus* accessions fell into four main clusters. Besides, this study suggested that geographical distance and environmental variables (annual mean precipitation and average precipitation in growing seasons), especially for QTP accessions, should be combined to explain the population genetic differentiation among the divergent geographical regions. These data provided comprehensive information about these valuable *E. excelsus* germplasm resources for the protection and collection of germplasms and for breeding strategies in areas of Xinjiang and QTP in western China.

## Introduction

*Elymus* Linn. is the most diverse and largest genus in Triticeae, with approximately 12 species widely distributed in northern China (Yan et al., 2005). Due to its close phylogenetic relationship with important cereal crops (eg. wheat, barley and rye), this genus has the considerable potential for improving cereal crops (Yan et al., 2005). As a perennial allohexaploid forage grass, *Elymus excelsus* Turcz. ex Griseb. possesses great tolerance to cold, drought and soil impoverishment and has a high seed yield; therefore, this species can be used for grassland improvement (Qi et al., 2009). In addition, *E. excelsus* constitutes an *E. dahuricus* complex along with *E. ivoroschilowii* Probat., *E. tangutorum* (Nevski) Hand.-Mazzand. and *E. duhuricus* Turcz. ex Griseb. (Agafonov et al., 2001). The genome composition of *E. excelsus* is StHY, in which the H and St are derived from *Hordeum* and *Pseudoroegneria*, respectively, while the ancestor of the Y genome is still unknown (Song, Nan & Tian, 2015). To date, most of the available investigations about *E. excelsus* mainly involve analysis of its taxonomic classification, physiological features and cultivation (Qi et al., 2009), and little or no research has been conducted regarding its germplasm collection and genetic variability.

Understanding genetic diversity and the relationship between wild germplasms in distinct ecogeographical conditions is essential for collection, sustainable management, effective conservation, and genetic improvement of germplasm resources (Shen & Liu, 2001). Having a complex geography and landscape and an obviously changeable climate, the Qinghai-Tibet Plateau (QTP), also known as the Roof of the World, and Xinjiang, as the heart of the Eurasian continent, are the major distribution areas of perennial Triticeae grasses (Wang & Li, 1993). Substantial attention has been paid to the collection, conservation and evaluation of plant species diversity in the QTP and Xinjiang areas that are suffering from natural habitat destruction, which has been caused by climate extremes and long-term grazing pressure (Wang & Li, 1993; Wang, 2011).

Morphological traits (Yan, Zhou & Wang, 2016), allozymes (Yan, Zhou & Wang, 2016), DNA markers (Zhang et al., 2018) and emerging sequencing technology can be used or have shown potential for evaluating the genetic diversity of *Elymus* L. Sequencing technology can identify suitable numbers of mutated loci with wide genome coverage and, thus, has been used in the evaluation of the genetic diversity of population germplasms (Li et al., 2015). For allopolyploid species with large genomes, however, it is not only difficult to perform bioinformatics analysis but also expensive. Molecular markers have been considered as powerful tools for characterizing plant genetic resources due to their being unaffected by phenotype and having a high discriminatory power and a comparatively low cost. At present, molecular markers, such as random amplified polymorphic DNA (RAPD) (Ma et al., 2009), inter-simple sequence repeat (ISSR) markers (Li et al., 2005), amplified fragment length polymorphisms (AFLPs) (Wu et al., 2019) and simple sequence repeats (SSRs) (Chen, 2013), have shown considerable potential for the identification of the genetic divergence in *Elymus* species. In particular, SSRs or microsatellite markers are often chosen for genetic mapping, gene tagging and genetic diversity research due to their specific PCR amplification, high polymorphic information content (PIC), good generalizability, high mutation rate, co-dominant inheritance and suitability for automated allele sizing (Fang & Li, 2018). In most studies on genetic diversity, the effect of SSRs is on par with SNP markers, and both of these markers can correctly reflect the true results (Li et al., 2015). In recent years, a large number of EST-SSRs and G-SSRs (genomic SSRs) have been developed from several *Elymus* species and possess high transferability across their related species (Chen, 2013; Luo et al., 2015; Bushman, 2008; Lei et al., 2014). However, there are no SSR markers developed from *E. excelsus*, and extremely limited information about the SSR diversity in *E. excelsus* accessions has been acquired.

The existing data show that wide hybridization, geographical isolation or local adaptations (such as altitude, precipitation and temperature) will promote the genetic diversity of the *Elymus* species (Yan et al., 2005). A combination of molecular markers plus ecogeographical data analysis can provide comprehensive inclusion of the utmost genetic diversity in plant germplasm collections. In previous studies about the genetic diversity of *E. nutans* and *E. sibiricus* accessions, however, the association between eco-geographical factors and genetic variability were ignored (Zhang et al., 2018). Here, 25 wild accessions of *E. excelsus* were used to perform a diversity analysis using both EST-SSRs and genomic SSRs, with the objectives of (i) evaluating the transference of EST-SSRs and G-SSRs developed from related species of Triticeae to *E. excelsus* accessions and the discrimination power of the tested accessions; (ii) determining the genetic diversity and population structure of the wild accessions; and (iii) assessing the effects of climatic and geographical variables on genetic diversity and population structure.

**Figure 1:**
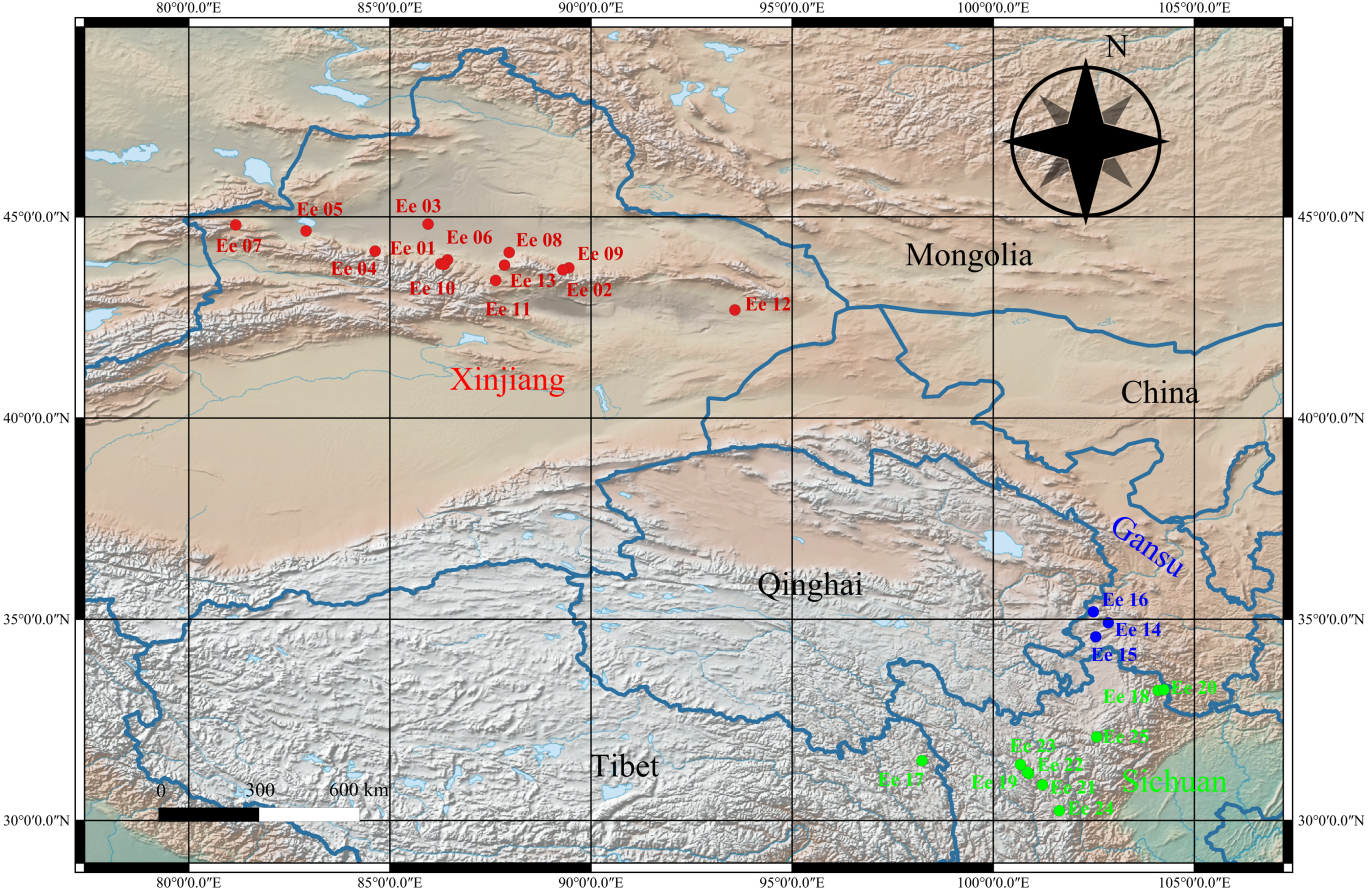
Geographical distribution of the studied * Elymus excelsus* accessions from western China.

## Materials & Methods

### Plant materials and DNA isolation

A wide collection including 25 *E. excelsus* wild accessions (Data S1, Table S1) was used in this study, and these accessions were collected from Xinjiang and the Qinghai-Tibet Plateau (Gansu, Sichuan and Tibet) in China. The accessions were assembled from the collections in the National Plant Germplasm System of USDA (NPGS) (with the prefixes ‘PI’) and Sichuan of China (with the prefixes “Elymus”) with the permission of the Sichuan Forestry and Grassland Bureau (Table S1). The QGIS software 3.6.1 (https://www.qgis.org/en/site/) was used to georeference the *E. excelsus* accessions on a map (Fig. 1) and its elevation layer was freely available from the Natural Earth (https://www.naturalearthdata.com). Ten seeds per accession of *E. excelsus* were placed in the culture dishes to germinate under uniform conditions in an incubator. Total DNA was isolated from the bulked young leaf tissues of each accession using a DNA extraction kit (Tiangen, Beijing). The concentration of each DNA sample was measured using a NanoDrop1 ND-1000 Spectrophotometer (NanoDrop Technologies, USA), and then, the extracted DNA was diluted to approximately 20 ng µL^−1^ for SSR analysis.

### SSR analysis

One hundred and twenty-six SSR primer pairs were used in the pre-experiment, including 98 EST-SSRs and 28 G-SSRs, in which 30 EST-SSRs and five G-SSRs primers, respectively, showed polymorphisms among 25 accessions and were selected for further research. The EST-SSRs were developed from *E. sibiricus* (with the prefixes ‘ES’, Table S2) (Chen, 2013), *E. nutans* (with the prefixes ‘Cn’) (Luo et al., 2015) and *Pseudoroegneria spicata* (with the prefixes ‘Elymus’) (Bushman, 2008), and the G-SSRs were developed from *E. sibiricus* (with the prefixes ‘ESGS’) (Lei et al., 2014). Following the protocol of Gu et al. (2015) with minor modifications, the SSR-PCR amplifications were carried out in a total of 15 µL reaction volumes including 3 µL (20 ng µL^−1^) template DNA, 0.8 µL (5 pmol µL^−1^) forward and reverse primers, 7.5 µL mix (containing 10 × PCR buffer, Mg^2+^, dNTPs), 0.4 µL Taq enzyme (2.5 U µL^−1^) (Beijing Tiangen Science and Technology Biochemical Company) and the remaining volume was supplemented with ddH_2_O. A PCR program was used with the following cycling parameters: first pre-denaturation step at 94 °C for 4 min, followed by 35 cycles of 30 s at 94 °C, annealing for 30 s at 51–66 °C (Table S2), 1 min at 72 °C and a final extension of 10 min at 72 °C. The amplified products were detected on an 8% non-denaturing polyacrylamide gel (acrylamide: methylene=19:1) with 1 × TBE buffer solution.

### Data acquisition and analysis

The patterns at all SSR loci were converted into a binary matrix that was recorded as 1/0 (presence of band/absence of band) for further study owing to the difficulty of unambiguously ascertaining allele dosage in allohexaploid. Based on the number of polymorphic bands (NPB), which was estimated according to the band frequency of less than 95% and more than 5%, the discriminatory power of each SSR marker was evaluated by computing the percentage of polymorphic bands (PPB), Shannon diversity index (I) (Wu et al., 2019), polymorphic information content (PIC) (Gu et al., 2015), marker index (MI), Nei’s genetic diversity (H) and resolving power (Rp). The MI was calculated as proposed by Powell et al. (1996) to reflect the polymorphism information of each pair of primers: MI=PIC ×NPB. The PIC of each amplified fragment was defined as follows, supposing that p and q, respectively, represent the frequency with band and the frequency without band: PIC =1-*q*^2^-*p*^2^ (Gu et al., 2015). Nei’s genetic diversity (H) and Shannon diversity index (I) were calculated using the GenAlEx 6.51 program (Peakall & Smouse, 2012). The Rp reflects the ability of a single pair of primers to differentiate genotypes in germplasm panels, and the calculating formula is: *Rp* = Σ Ib, where the calculating formula of Ib, which represents a single strip of information (band informativeness) is: Ib = 1-(2 × |0.5 − *P*_*i*_|), where *P*_*i*_ is the amplification bands frequency in the studied accessions (Gu et al., 2015). Finally, the significance of the difference between the PIC values derived from the EST-SSR vs. G-SSR data were assessed by the Wilcoxon rank sum test using SPSS 19.0 software (SPSS Inc., Chicago, IL, USA).

Based on DICE coefficient (Dice, 1945), a binary matrix was further used to calculate the genetic similarity (GS) between pairs of accessions using FREETREE software (Pavlícek, Hrdá & Flegr, 1999). Then, principal coordinates analysis (PCoA) and the Unweighted Pair Group Method with Arithmetic (UPGMA) method were performed. The dendrogram robustness was tested with bootstrap values (1,000 substitutions) via Fig Tree V 1.4.3 software (Hampl, Pavlicek & Flegr, 2001). The degree of genetic variation among and within geographical groups was analysed using the non-hierarchical analyses of molecular variance (AMOVA) method, with 9,999 times random permutations (Peakall & Smouse, 2012). Furthermore, the GenAlex 6.51 program was also used to calculate the number of different alleles (N_a_), number of effective alleles (*N*_*e*_), Shannon’s information index (*H*_*j*_), expected heterozygosity (*H*_*e*_), Nei’s genetic distance and pairwise population PhiPT values (*F*_st_) among the geographical groups.

In addition, using STRUCTURE (Evanno, Regnaut & Goudet, 2005), a model-based Bayesian clustering program, the membership of each accession was characterized with a range of genetic clusters from *K* = 2 to 10. Four independent runs were assessed for each fixed K and each run consisted of a ”Burnin Period” of 50,000 and “After Burnin” MCMCC (Markov Chain Monte Carlo) replicates of 10,0000. The optimal potential clusters (K value) were determined by the statistic ΔK method on the SRTUCTURE Harvester v.0.6.93 program (Evanno, Regnaut & Goudet, 2005). The running results were integrated by CLUMPP1.1 software (Jakobsson & Rosenberg, 2007) and were visualized using the GraphPad Prism 5 program (GraphPad software, San Diego, CA).

On the basis of the geographical distributions of the studied accessions, the climatic variables, such as average annual precipitation, average annual temperature, elevation and mean rainfall during the growing season (from May to August) obtained from DIVA-GIS (version 5.2.0.2, http://www.diva-gis.org/) were used to evaluate the association with genetic distance (Table S1). Geographic Distance Matrix generator_v1.2.3 software was used to calculate the geographical distance matrix, and then the correlation between the genetic differentiation and geographical distance or environmental differences were calculated by a Mantel test using NTSYS-PC software v2.02 (Exeter software, New York) and visualized in R package version R 3.4.1.

## Results

### SSR polymorphism and genetic diversity in the germplasm collection

In total, 35 SSR primer pairs, including thirty (30.61% out of 98) EST-SSRs and five (17.86% out of 28) G-SSRs, allowed the identification of 159 reliable polymorphic bands (NPB). The number of polymorphic bands (NPB) of each primer pairs changed from one (ES 7, ES 75) to 14 (Elymus 5264) with an average number of 4.54. The total number of bands (TNB) amplified by each primer pairs ranged from four (ES 352, ES 176 and Cn 48) to 16 (Elymus 5264) with an average of 7.2 and a total of 252 (Table 1). The percentage of polymorphic bands (PPB) of each primer pairs changed from 11.11% (ES 75) to 100% (ES 352, ES 176, Cn 479) with an average of 61.37%. In addition, the PIC (ranged from 0.147 to 0.466), MI (ranged from 0.294 to 3.822), H (ranged from 0.078 to 0.480), I (ranged from 0.171 to 0.673) and Rp (ranged from 0.160 to 5.760) were recorded to evaluate the polymorphisms of the primers and their resolution in regards to the tested accessions. There was positive association between the MI and Rp (*r* = 0.972, *P* < 0.001), and both PIC and H were significantly correlated with I (*r* = 0.263 and 0.997, respectively, *P* < 0.001). It is noteworthy that the results revealed a higher transferability of the EST-SSR (30.61%) than the G-SSR (17.86%), while all the average diversity parameters (TNB, NPB, PPB, PIC, Rp, MI, H and I) of the G-SSRs were higher than those of the EST-SSRs (Table 1); these results showed that the EST-SSR had higher generalizability than the G-SSR in *E. excelsus* accessions, but the amplified bands of the G-SSR were more polymorphic (measured as PIC) than those of the EST-SSR (0.373 vs 0.275, respectively, Wilcoxon rank sum test, *P* = 0.048).

**Table 1:** Marker parameters calculated for each SSR primer combination used with *E. excelsus* accessions.

Primers	Type	TNB	NPB	PPB (%)	PIC	MI	Rp	H	I
Cn 159	EST-SSR	8	3	37.50	0.205	0.615	0.72	0.224	0.368
Cn 193	EST-SSR	6	4	66.67	0.179	0.716	0.80	0.429	0.620
Cn 204	EST-SSR	5	2	40	0.355	0.710	1.20	0.428	0.619
Cn 227	EST-SSR	6	3	50	0.169	0.507	0.56	0.200	0.333
Cn 237	EST-SSR	13	9	69.23	0.188	1.692	1.92	0.323	0.483
Cn 278	EST-SSR	6	5	83.33	0.173	0.865	0.96	0.228	0.360
Cn 291	EST-SSR	5	3	60	0.303	0.909	1.12	0.381	0.554
Cn 294	EST-SSR	9	6	66.67	0.384	2.304	3.52	0.345	0.520
Cn 299	EST-SSR	6	3	50	0.226	0.678	0.80	0.339	0.505
Cn 306	EST-SSR	5	2	40	0.147	0.294	0.32	0.406	0.596
Cn 350	EST-SSR	7	5	71.43	0.439	2.195	3.52	0.373	0.554
Cn 362	EST-SSR	5	3	60	0.169	0.507	0.56	0.312	0.470
Cn 479	EST-SSR	6	6	100	0.361	2.166	2.96	0.431	0.611
Cn 48	EST-SSR	4	2	50	0.211	0.422	0.48	0.116	0.232
Elymus 2644	EST-SSR	5	2	40	0.24	0.480	0.56	0.135	0.260
Elymus 3207	EST-SSR	15	13	86.67	0.32	4.160	5.76	0.367	0.543
Elymus 3592	EST-SSR	7	5	71.43	0.279	1.395	1.76	0.279	0.430
Elymus 5264	EST-SSR	16	14	87.50	0.273	3.822	4.96	0.320	0.482
ES 105	EST-SSR	8	5	62.50	0.383	1.915	3.12	0.390	0.570
ES 123	EST-SSR	9	4	44.44	0.238	0.952	1.12	0.380	0.557
ES 176	EST-SSR	4	4	100	0.338	1.352	2.08	0.287	0.440
ES 179	EST-SSR	5	2	40	0.458	0.916	1.44	0.319	0.499
ES 180	EST-SSR	6	3	50	0.382	1.146	1.84	0.337	0.503
ES 261	EST-SSR	5	2	40	0.147	0.294	0.32	0.078	0.171
ES 322	EST-SSR	8	4	50	0.147	0.588	0.64	0.078	0.171
ES 352	EST-SSR	4	4	100	0.226	0.904	1.20	0.221	0.359
ES 51	EST-SSR	9	4	44.44	0.466	1.864	2.96	0.356	0.540
ES 7	EST-SSR	7	1	14.29	0.147	0.147	0.16	0.078	0.171
ES 75	EST-SSR	9	1	11.11	0.461	0.461	0.72	0.480	0.673
ES 82	EST-SSR	5	3	60	0.228	0.684	0.80	0.346	0.519
ESGS 124	G-SSR	5	3	60	0.45	1.35	2.24	0.464	0.657
ESGS 172	G-SSR	8	7	87.50	0.318	2.226	3.12	0.203	0.347
ESGS 266	G-SSR	10	9	90	0.385	3.465	5.28	0.308	0.474
ESGS 292	G-SSR	10	8	80	0.309	2.472	3.60	0.212	0.351
ESGS 52	G-SSR	6	5	83.33	0.404	2.02	3.28	0.365	0.536
Min	–	4	1	11.11	0.147	0.294	0.16	0.078	0.171
Max	–	16	14	100	0.466	3.822	5.76	0.480	0.673
Mean	–	7.2	4.54	61.37	0.289	1.348	1.897	0.301	0.459
Mean (EST-SSR)	–	7.1	4.23	58.24	0.275	1.189	1.629	0.300	0.457
Mean (G-SSR)	–	7.8	6.4	80.17	0.373	2.307	3.504	0.310	0.473

**Notes.**

PIC polymorphic information content TNB the total number of bands NPB the number of polymorphic bands PPB the percentage of polymorphic bands MI marker index Rp resolving power I Shannon diversity index H heterozygosity

### Clustering and population structure analysis

**Figure 2:**
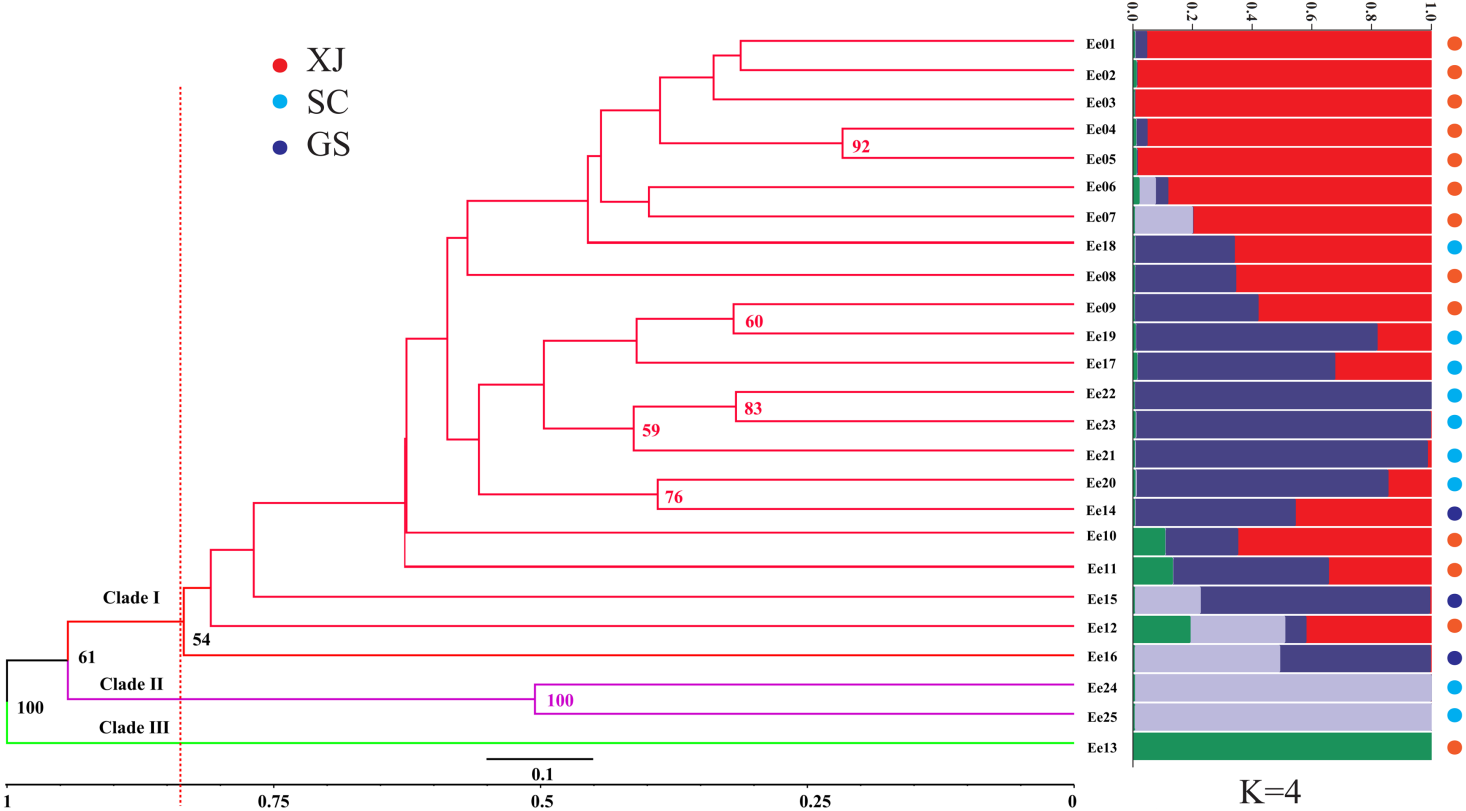
Unweighted Pair Group Method with Arithmetic (UPGMA) tree of *Elymus excelsus* wild accessions and genetic relationship among *E. excelsus* accessions using a Bayesian analysis of the geo-group structure at *K* = 4.

The genetic similarity among the tested accessions ranged from 0.6667 to 0.9496, which were calculated based on the 252 amplified fragments (supplementary data, Table S3). Two accessions from Sichuan (SCC, Ee 22 and Ee 25) were the most divergent due to a minimum genetic similarity of 0.6667, while two accessions from Xinjiang (XJC, Ee 04 and Ee 05) were the most related with a maximum genetic similarity of 0.9496. An unrooted UPGMA dendrogram was constructed, and it divided 25 accessions into three clades according to their average genetic similarity value (0.8346) (Clade I, Clade II and Clade III, Fig. 2). Clade I included one accession from Gansu (GSC), and Clade II consisted of two accessions from SCC, while all of the rest of the accessions belonged to Clade III. The hierarchical clustering results were approximately consistent with PCoA, and the over 50% bootstrap support value of each main branch of the dendrogram indicated the reliability of the clustering results.

STRUCTURE software was used to assess the population stratification of the studied accessions based on a Bayesian model. According to Evanno’s method, the best K value was four in the present study (Fig. S1), which indicated that the studied accessions belonged to four memberships (Fig. 2). Supposing that membership proportion of 0.8 or more are treated pure (Forsberg et al., 2015), the pure frequency of the SCC group (66.67%) was the highest while two accessions (Ee 23 and Ee 24) of SCC had completely different genetic memberships compared with the other SCC accessions.

### PCoA

**Figure 3:**
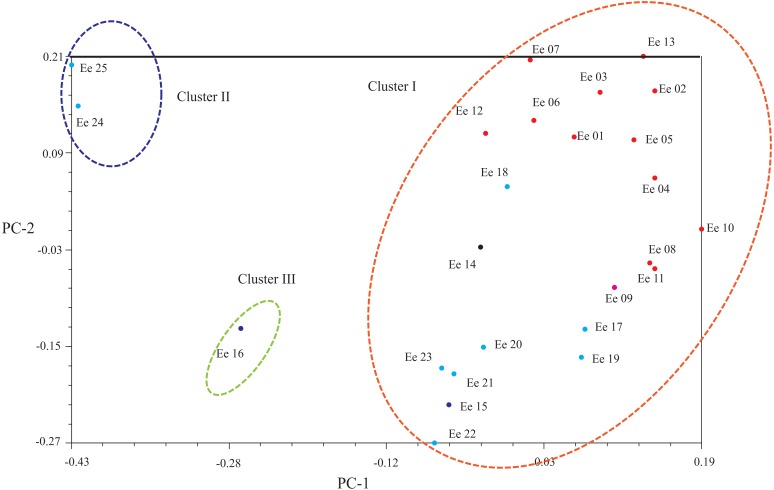
Principal coordinate analysis (PCoA) showing the relationships of the *Elymus excelsus* accessions.

Another clustering pattern of the tested accessions was carried out by principal coordinates analysis (PCoA, Fig. 3), which demonstrated that a scatter-plot depiction of PCoA was equivalent to both the hierarchical and Bayesian cluster analysis. In short, most of the accessions could put into the same large cluster. Based on Dice’s distances, PCoA confirmed the division of the 25 accessions into three clusters: Clusters I, II and III. Principal component one (PC1) explained 13.94% of the molecular variation, which separated most accessions, and principal component two (PC2) explained 12.00% of the variation, which could be further distinguished from the accessions from Clusters I and III.

### Genetic structure of the geographic groups and their diversity indexes

All *E. excelsus* accessions were divided into three geo-groups: XJC (Xinjiang, 13), SCC (Sichuan, 9, including one from Tibet) and GSC (Gansu, 3) based on geographical origin, in which the SCC group displayed the highest level of variability (*N*_*a*_ = 1.444, *N*_*e*_ = 1.340, *H*_*j*_ = 0.297 and *H*_*e*_ = 0.197, Table 2). The intra-group genetic diversity was highest within the SCC group (*H*_*e*_ = 0.197) followed by the XJC and GSC groups (*H*_*e*_ = 0.148 and 0.176, respectively). AMOVA was used to test the influence of the geographic origin on the genetic diversity of the *E. excelsus* accessions. Of the total genetic variance covered by the three geo-groups, 15% (Table 3) was due to the variation among geographic groups while 85% of the variation was due to variation among accessions within geographic groups. Moreover, the differences within the groups and among the groups were all statistically significant (*P* < 0.01). The average fixation index (*F*_st_) among the three groups demonstrated a moderate genetic differentiation (*F*_st_ = 0.151). Otherwise, the pairwise *F*_st_ value between the XJC and GSC groups was the highest (0.297, Table S4) while the value between the SCC and GSC groups was the lowest (0.012, Table S4).

**Table 2:** Different genetic diversity estimates for three geographical groups of *E. excelsus* accessions.

Geographical Group	N	N_a_	N_e_	H_j_	H_e_	uH_e_	*P*
XJC	13	1.313 ± 0.048	1.249 ± 0.022	0.226 ± 0.017	0.148 ± 0.012	0.154 ± 0.012	64.39%
SCC	9	1.444 ± 0.047	1.340 ± 0.024	0.297 ± 0.018	0.197 ± 0.013	0.209 ± 0.013	51.62%
GSC	3	1.214 ± 0.050	1.309 ± 0.024	0.259 ± 0.019	0.176 ± 0.013	0.211 ± 0.016	36.51%

**Notes.**

N Individual number of populations N_a_ No. of different Alleles N_e_ No. of effective alleles H_j_ Shannon’s information index H_e_ Expected heterozygosity uH_e_ Unbiased expected heterozygosity P Genetic variation

**Table 3:** Analysis of molecular variance (AMOVA) among and within geographical groups of *Elymus.* excelsus accessions.

Source of variation	df	PMV (%)	SS	MS	Est. Var.	*F*_st_	*P* value
Three groups							
Among groups	2	15	109.904	54.952	4.252	0.151	0.003
Within groups	22	85	524.256	23.830	23.830		
Total	24	100	634.160		28.081		

**Notes.**

df degree of freedom PMV Percentages of molecular variance SS square deviation MS mean square deviation Est. Var. exist variance *F*_st_ coefficient of genetic differentiation

### Genetic variation associated with environmental factors

**Figure 4:**
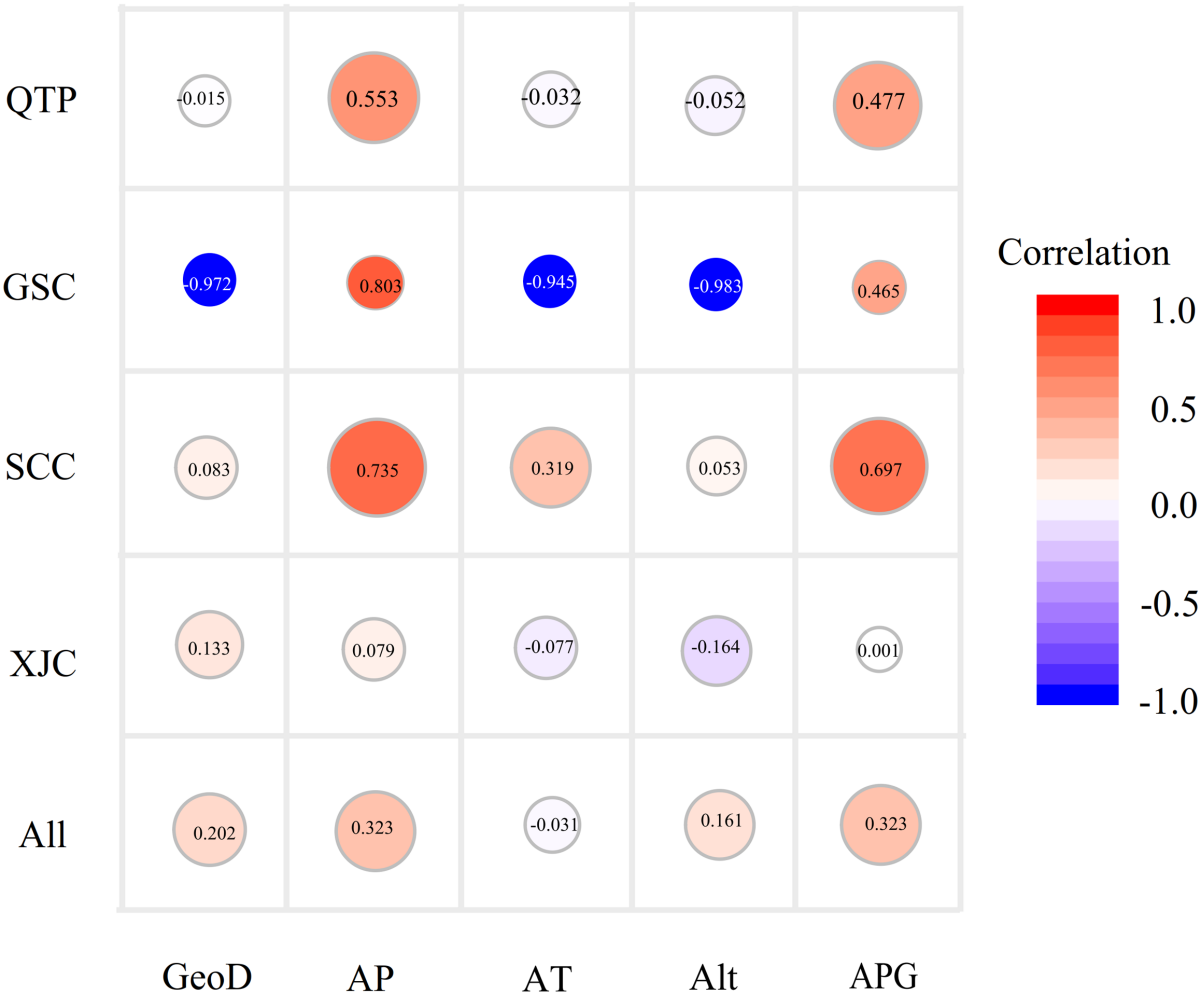
Bubble diagrams of the correlation between genetic distance and geo-environmental factors in species and geo-groups levels. GeoD, geographical distance; AP, mean annual precipitation; AT, mean annual temperature; Alt, altitude; APG, average precipitation of growing seasons. Correlation coefficients calculated by Mantel test are showing in bubbles.

Since Sichuan and Gansu provinces both belong to the QTP areas, here we amalgamated the SCC and GSC groups into a new group, namely, the QTP group. A Mantel test was carried out to explore the influence of geographical distance on genetic variation, which demonstrated a faint but significantly positive correlation between genetic distance and geographical (*r* = 0.202, *P* < 0.01; Fig. 4) or altitude distance (*r* = 0.1614, *P* < 0.05) at the species level (Table S5). A similar result was observed in the matrix correlation between the average annual precipitation distance and the genetic distance (*r* = 0.323, *P* < 0.01; Fig. 4). In addition, a non-significant association between genetic distance and four environmental distances as shown in Fig. 4 were observed, except for the annual mean precipitation and mean rainfall of the growing seasons (from May to August) in the SCC accessions (*r* = 0.697 and *r* = 0.735, respectively, *P* < 0.01) and the QTP groups (*r* = 0.553 and *r* = 0.476, *P* < 0.05). Furthermore, a relatively high but non-significant Mantel relationship value between the genetic distance and all of the studied environmental factors were detected in the GSC accessions, which may be due to the small group size (only three accessions).

## Discussion

### SSR polymorphisms and the discriminating capacity of the assays

To better characterize the population genetic diversity in different geographical locations, SSR molecular markers were used to identify the genetic differentiation of *E. excelsus* accessions at the individual and population levels (based on geographical origins). The results showed that SSR markers could be used to investigate the genetic relationships among wild *E. excelsus* accessions, and 61.37% of the polymorphic loci were observed, which was higher than that of *E. sibiricus* (50.7%) (Ma et al., 2009) but lower than that of *E. nutans* (79.75%) (Chen, 2013).

The PIC refers to the discriminatory power or informativeness of markers and has been extensively applied in animals and plants (Gu et al., 2015). The MI and Rp, two important parameters in choosing polymorphic markers, have been widely applied in diversity studies (Powell et al., 1996). I (the Shannon diversity index) represents an authentic alternative measure of diversity because there is no need for an estimate of the allele frequencies under a Hardy-Weinberg equilibrium (Wu et al., 2019). The average PIC value of 0.289 in the present study showed excellent marker discriminatory ability in light of the PIC value ranging from 0 to 0.5 for the dominant marker (Varshney et al., 2007). According to the study of Gupta et al. (2013), the identification ability of the primers has a strong correlation with the Rp and MI values. The average Rp (1.897) and MI value (1.348) of the primers used in this investigation were lower compared to those observed by Najaphy et al. (2013) (Rp = 12) and Li et al. (2005) (MI = 4.3) using ISSR and SSR markers, respectively. The average PIC, MI and Rp per primer pair suggested that the 35 SSRs developed from related species of the Triticeae (*E. sibiricus*, *E. nutans* and *Pseudoroegneria spicata*) and could also be used to distinguish *E. excelsus* accessions. If the PIC, MI and Rp can be used as indicators to characterize the effectiveness of accessions, Elymus 3207 (PIC = 0.320, MI = 4.160, Rp = 5.760), Elymus 5264 (PIC = 0.273, MI = 3.822, Rp = 4.960) and ESGS 266 (PIC = 0.385, MI = 3.465, Rp = 5.280) are recommended as the ideal SSR primers.

Due to the higher sequence conservation in the coding sequences compared to the non-coding sequences, EST-SSRs have generally lower allele numbers and higher transferability than genomic SSRs in related species (Yan et al., 2007). For instance, the EST-SSRs used here had higher generality (30.61%) for *E. excelsus* accessions than G-SSRs (17.86%), which was in accordance with the study results for *E. nutans* (Meyer et al., 2017). The present study found that the EST-SSRs and G-SSRs developed from *E. nutans*, *E. sibiricus* and *P. spicata* were successfully applied to the identification of the genetic diversity of *E. excelsus* accessions. The EST-SSRs were more versatile while the G-SSRs were more effective, so the combination of the EST-SSRs and G-SSRs is strongly proposed to characterize the genetic variation of plant germplasm (Meyer et al., 2017).

### Clustering pattern and genetic structure

The accurate exploration of the genetic relationships of germplasm is significant for resource conservation, evolutionary research and cultivar development (Najaphy, Parchin & Farshadfar, 2011). Three clusters were identified for the 25 accessions by the UPGMA and PCoA methods. Except for three accessions, all of the accessions were grouped into one large cluster. The possible reason for such a clustering pattern may be high levels of gene flow between Sichuan and Gansu accessions, due to geographical proximity, which was confirmed by mixed genealogies of the SCC and GSC accessions using the STRUCTURE program. It is worth noting that most of the XJC accessions were clustered together with three GSC and seven SCC accessions. This result, which demonstrated that geographical isolation did not always lead to greater genetic differentiation, is consistent with the study of the related *E. nutans*, a self-pollinated hexaploid species (Chen et al., 2010). Exceptions to these clustering patterns were accessions Ee 24, Ee 25 (both from SCC) and Ee 13 (from XJC), which were separated from the other accessions and grouped into Clade II and Clade III, respectively. Because *E. excelsus* could be used for reseeding in grassland improvement of western China (Qi et al., 2009), the possible reason was, to a great extent, that the three accessions mentioned above were not indigenous and might have been introduced from other localities, which is why accessions Ee 24 and Ee 25 had a minimum genetic similarity of 0.6667.

Clustering by genetic distance usually produces only exploratory results (Falush, Stephens & Pritchard, 2007). To accurately explore the genetic structure of *E. excelsus* accessions, STRUCTURE software was used to perform further analysis based on a Bayesian model, showing four potential genetic backgrounds. In comparison, in the figures of the UPMGA and STRUCTURE analysis, five SCC accessions (Ee 19, Ee 20, Ee 21, Ee 22 and Ee 23) in Clade I of the UPGMA clustering had obviously distinct genome fractions in the STRUCTURE analysis. That result may be due to the vulnerability of the STRUCTURE program to influences, including gene drift, gene mutation, gene flow and natural selection, etc. (Evanno, Regnaut & Goudet, 2005).

The high intra-group variation components (85%) among the three geo-groups might be attributable to the dominantly self-pollinated breeding system of *E. excelsus* and sampling at a large spatial scale (Nybom, 2004). Due to the geographical isolation via barriers, such as rivers and mountains, each accession of *E. excelsus*, which could be considered as a population constituting seeds collected from adjacent grasses of distinct locations (Reed et al., 2004), will maintain their own internal characteristics and show low within-accession variability. Therefore, it’s not surprising that the most of the variability of proportions investigated in the *E. excelsus* accessions in the present study existed within groups at a large-scale geographical level containing a great number of accessions. Meanwhile, the genetic variation within the SCC group (H_e_ = 0.197) was greater than that of the GSC (H_e_ = 0.176) and XJC groups (H _e_ = 0.148); therefore, it was further inferred that a high variable climate and complex topography might increase the genetic diversity of accessions from the southeast boundary of the QTP (Wang & Li, 1993), which has a typical plateau mountain climate with higher average annual precipitation than in the other groups. Further analysis of the genetic variability and climatic factors by the Mantel test were as follows.

### Correlation between genetic variability and environmental factors

Owing to divergent selection and local adaptation, spatial environmental variation and ecological diversification between habitats are essential for the preservation of genetic diversity (Dawson et al., 2011). The present study was the first comprehensive evaluation of a correlation between genetic variability and the environmental diversity of wild *E. excelsus* accessions. We also found a weak geographic signature of isolation-by-distance (*r* = 0.202, *P* < 0.01) among the tested *E. excelsus* accessions, which is similar to a study of the related *E. nutans* (Chen, 2013). However, there was no significant relationship between geographical distance and the population structure of *E. glaucus* germplasm accessions from Oregon (Wilson et al., 2001), which might be caused by the differences of habitat and even types of molecular markers used between those two studies. Nevertheless, there are limitations in the use of geographical distance solely for reflecting the spatial distribution of genetic diversity, which is influenced by both complex topographic features and climates (Falush, Stephens & Pritchard, 2007). Therefore, the relationship between environmental divergence and genetic distance within geographical groups were further analysed. This study did not find similar results to previous research that indicated a correlation between altitude and the genetic distance of *Elymus* species (Blanco et al., 2016), but there was a significant correlation between the mean rainfall during the growing season, the average annual precipitation and the genetic distance of the QTP geo-group; this result suggested that precipitation might contribute to the hierarchical structure of the accessions from Sichuan (SCC) with its high rainfall and high altitude accompanied by high evaporation (which means higher precipitation is demanded).

## Conclusions

This study indicated that SSR markers developed from related species are powerful tools to characterize the genetic diversity of wild *E. excelsus* accessions, and geo-climatic variabilities play a significant part in genetic divergence of geographical groups. Three hierarchical structure analyses together revealed a genetic heterogeneity of *E. excelsus* genotypes, especially for the QTP group, and the geographic complexity and climatic diversity of Xinjiang and QTP (Qinghai-Tibet Plateau) were emphasized. All results emphasize the significance of local adaptation in the forming of the models of *E. excelsus* wild accessions, and these findings are beneficial to protection strategies and utilization of germplasm resources.

##  Supplemental Information

10.7717/peerj.8038/supp-1 Data S1 Binary matrix that recorded as 1/0

10.7717/peerj.8038/supp-2 Figure S1 Estimation of number of subpopulations (K value) for the *E. excelsus* accessions from STRUCTURE analysis

10.7717/peerj.8038/supp-3 Table S1 The eco-geographic description on collection sites of *Elymus excelsus* accessions MAT, Mean annual temperature; MAP, Mean annual precipitation; MP (May to August), Mean rainfall from May to August.

10.7717/peerj.8038/supp-4 Table S2 List of primers, their sequences, the annealing temperature and their references Ta represented the annealing temperature; references were quoted from: [1] Lei Y, Zhao Y, Yu F, et al. Development and characterization of 53 polymorphic genomic-SSR markers in Siberian wildrye (*Elymus sibiricus* L.). Conservation Genetics Resources, 2014, 6(4): 861-864. [2] Zhou Q, Luo D, Ma LC, Xie WG, Wang Y, Wang YR, Liu ZP. Development and cross-species transferability of EST-SSR markers in Siberian wildrye (*Elymus sibiricus* L.) using Illumina sequencing. SCI REP. 2016;6:20549. [3] Bushman BS. Development and annotation of perennial Triticeae ESTs and SSR markers. Genome. 2008;51:779-788. [4] Luo D, Zhou Q, Ma LC, Xie WG, Wang YR, Hu XW, Liu ZP. Novel polymorphic expressed-sequence tag–simple-sequence repeat markers in *Campeiostachys nutans* for genetic diversity analyses. Crop Sci. 2015;55:2712-2718.

10.7717/peerj.8038/supp-5 Table S3 Genetic similarity matrix of* Elymus. excelsus* accessions based on DICE coefficient and REETREE software

10.7717/peerj.8038/supp-6 Table S4 Pairwise Population PhiPT Values among XJ, SC and GS groups of* E. excelsus* accessions

10.7717/peerj.8038/supp-7 Table S5 Genetic distance matrix and geo-environmental distance matrixes used for Mantel test

## Additional Information and Declarations

### Field Study Permissions

The following information was supplied relating to field study approvals (i.e., approving body and any reference numbers): The collection of plant samples was conducted with the permission of the Sichuan Forestry and Grassland Bureau (Sichuan, China)

### Data Availability

The following information was supplied regarding data availability: The raw data is available at Zenodo: Yanli Xiong. (2019). microsatellite data [Data set]. Zenodo. http://doi.org/10.5281/zenodo.3502872
