# Supplementary figures and images for: Revelation of genetic diversity and structure of wild Elymus excelsus (Poaceae: Triticeae) collection from western China by SSR markers
Source: PeerJ. 2019 Nov 12;7:e8038. doi: 10.7717/peerj.8038 (PMC6857585; doi:10.7717/peerj.8038)

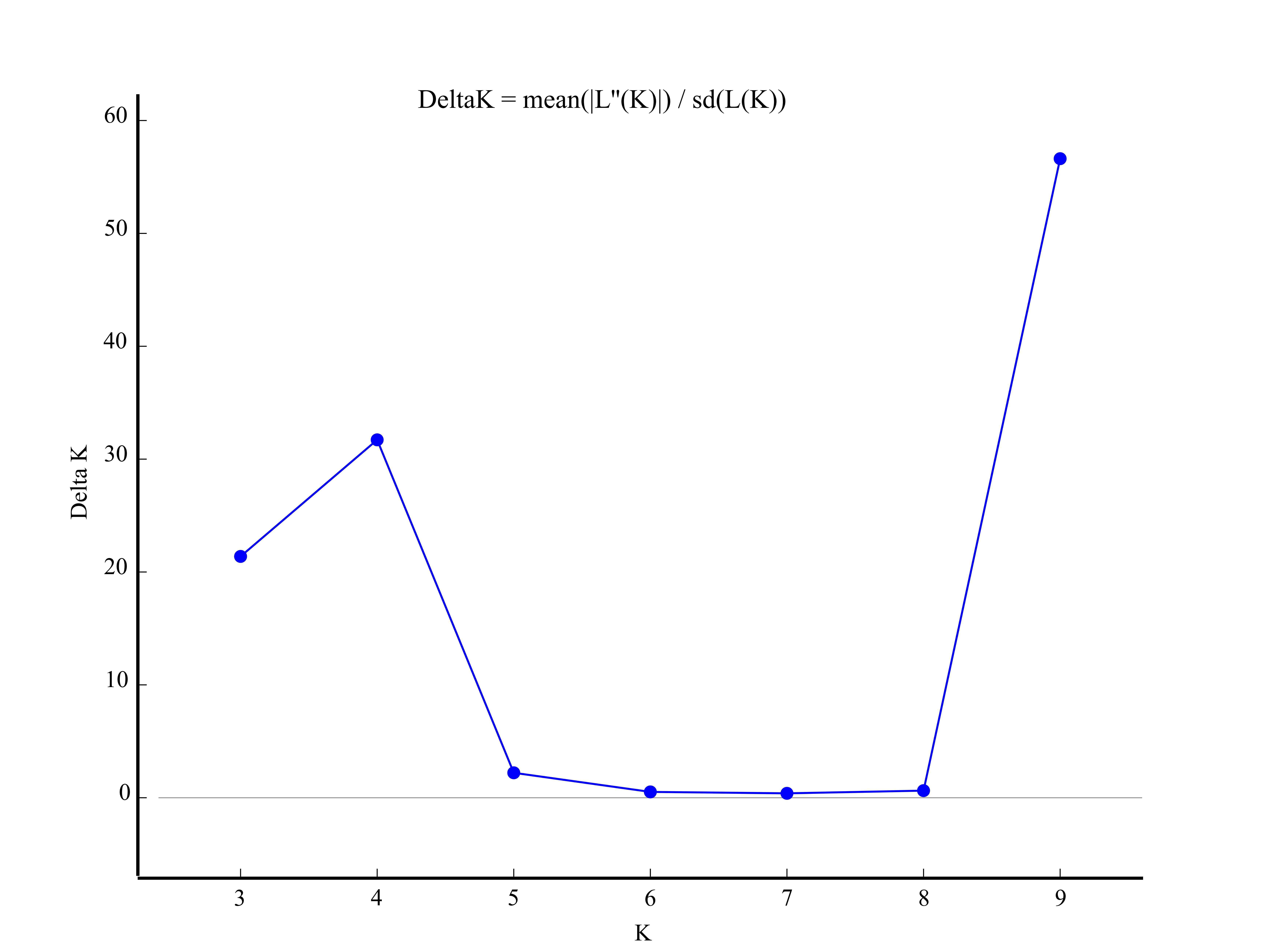

Supplement: Figure S1 [file peerj-07-8038-s002.png]
