# Supplementary material for: Revelation of genetic diversity and structure of wild Elymus excelsus (Poaceae: Triticeae) collection from western China by SSR markers
Source: PeerJ. 2019 Nov 12;7:e8038. doi: 10.7717/peerj.8038 (PMC6857585; doi:10.7717/peerj.8038)
Supplement: Table S1 — MAT, Mean annual temperature; MAP, Mean annual precipitation; MP (May to August), Mean rainfall from May to August. [file peerj-07-8038-s003.docx]

| No. | Accessions | Collection regions | Latitude (N) | Longitude (E) | Elevation (m) | MP (May to August)（mm） | MAT (℃) | MAP (mm) |
| --- | --- | --- | --- | --- | --- | --- | --- | --- |
| Ee 01 | PI 598528 | Changji co., Xinjiang, XJC | 43°49′59" | 86°16′0" | 1665 | 45.5 | 1.633 | 255 |
| Ee 02 | PI 598564 | Changji co., Xinjiang, XJC | 43°40′59" | 89°17′59" | 1870 | 29.5 | 2.108 | 195 |
| Ee 03 | PI 598536 | Shihezi city, Xinjiang, XJC | 44°49′12" | 85°57′0" | 1220 | 17 | 9.633 | 129 |
| Ee 04 | PI 598537 | Tacheng Area, Xinjiang, XJC | 44°8′59" | 84°37′59" | 1620 | 45 | 0.083 | 257 |
| Ee 05 | PI 598555 | Bortala co., Xinjiang, XJC | 44°38′59" | 82°55′0" | 300 | 13 | 8.167 | 108 |
| Ee 06 | PI 598530 | Changji co., Xinjiang, XJC | 43°55′59" | 86°25′59" | 1040 | 27.75 | 7.242 | 186 |
| Ee 07 | PI 598552 | Bortala co., Xinjiang, XJC | 44°47′59" | 81°10′0" | 1700 | 58.25 | 0.696 | 426 |
| Ee 08 | PI 598558 | Changji co., Xinjiang, XJC | 44°7′0" | 87°58′0" | 1680 | 21.25 | 7.688 | 188 |
| Ee 09 | PI 598561 | Changji co., Xinjiang, XJC | 43°43′59" | 89°27′0" | 1400 | 25.50 | 3.421 | 176 |
| Ee 10 | PI 610896 | Changji co., Xinjiang, XJC | 43°49′0" | 86°20′59" | 1510 | 42.75 | 2.704 | 244 |
| Ee 11 | PI 655139 | Urumqi co., Xinjiang, XJC | 43°49′12" | 87°36′35.99" | 1320 | 27.75 | 6.900 | 234 |
| Ee 12 | PI 619524 | Hami city, Xinjiang, XJC | 42°41′10" | 93°34′44" | 1750 | 5.25 | 10.288 | 35 |
| Ee 13 | PI 598566 | Urumqi co., Xinjiang, XJC | 43°47′59" | 87°50′59" | 1600 | 34.25 | 3.513 | 241 |
| Ee 14 | PI 655189 | Gannan co., Gansu, GSC | 34°54′48" | 102°51′47" | 2900 | 96.75 | 2.133 | 585 |
| Ee 15 | PI 636678 | Gannan co., Gansu, GSC | 34°33′48" | 102°33′5" | 2950 | 101.25 | 1.550 | 615 |
| Ee 16 | PI 655187 | Gannan co., Gansu, GSC | 35°11′9" | 102°29′25" | 2830 | 96.5 | 1.050 | 573 |
| Ee 17 | PI 619588 | Jiangda co., Tibet, SCC | 31°29′14" | 98°13′52" | 3600 | 92.75 | 2.638 | 531 |
| Ee 18 | Elymus e01 | Jiuzhaigou co, Sichuan, SCC | 33°13′59" | 104°5′59" | 2100 | 111 | 4.358 | 741 |
| Ee 19 | Elymus e02 | Ganzi co., Sichuan, SCC | 31°10′0" | 100°52′59" | 3520 | 119.25 | 3.879 | 698 |
| Ee 20 | Elymus e03 | Jiuzhaigou co., Sichuan, SCC | 33°15′05" | 104°14′37" | 2480 | 97.5 | 8.525 | 644 |
| Ee 21 | Elymus e04 | Ganzi co., Sichuan, SCC | 30°53′12" | 101°13′40" | 3280 | 127 | 3.571 | 744 |
| Ee 22 | Elymus e05 | Ganzi co., Sichuan, SCC | 31°11′22" | 100°51′50" | 2990 | 111.25 | 6.083 | 658 |
| Ee 23 | Elymus e06 | Ganzi co., Sichuan, SCC | 31°23′25" | 100°40′42" | 2990 | 110.75 | 5.479 | 658 |
| Ee 24 | Elymus e07 | Ganzi co., Sichuan, SCC | 30°14′40" | 101°38′36" | 3500 | 150.25 | 2.633 | 879 |
| Ee 25 | Elymus e08 | Aba co., Sichuan, SCC | 32°4′42" | 102°34′22" | 3280 | 140 | 0.846 | 872 |
