# Supplementary material for: Revelation of genetic diversity and structure of wild Elymus excelsus (Poaceae: Triticeae) collection from western China by SSR markers
Source: PeerJ. 2019 Nov 12;7:e8038. doi: 10.7717/peerj.8038 (PMC6857585; doi:10.7717/peerj.8038)
Supplement: Table S2 — Ta represented the annealing temperature; references were quoted from: [1] Lei Y, Zhao Y, Yu F, et al. Development and characterization of 53 polymorphic genomic-SSR markers in Siberian wildrye (Elymus sibiricus L.). Conservation Genetics Resources, 2014, 6(4): 861-864. [2] Zhou Q, Luo D, Ma LC, Xie WG, Wang Y, Wang YR, Liu ZP. Development and cross-species transferability of EST-SSR markers in Siberian wildrye (Elymus sibiricus L.) using Illumina sequencing. SCI REP. 2016;6:20549. [3] Bushman BS. Development and annotation of perennial Triticeae ESTs and SSR markers. Genome. 2008;51:779-788. [4] Luo D, Zhou Q, Ma LC, Xie WG, Wang YR, Hu XW, Liu ZP. Novel polymorphic expressed-sequence tag–simple-sequence repeat markers in Campeiostachys nutans for genetic diversity analyses. Crop Sci. 2015;55:2712-2718. [file peerj-07-8038-s004.docx]

| Primers | Type | Primer sequence (5'-3') | Ta (℃) | Reference |
| --- | --- | --- | --- | --- |
| ESGS52 | G-SSR | F: TTAGGGAACATCACAAGGT | 51 | [1] |
|  |  | R: CCAACATAATGAAGTAGAG |  |  |
| ESGS292 | G-SSR | F: TTGGGTGTGATGACAAGG | 61 | [1] |
|  |  | R: GGGCAAAATGCTACGATG |  |  |
| ESGS266 | G-SSR | F: CACAGAGCGAGTTGGTT | 62 | [1] |
|  |  | R: TTGTCACGTTTGAAGCA |  |  |
| ESGS172 | G-SSR | F: TTGAAGCAAGTACAACTA | 57 | [1] |
|  |  | R: GTAAAATCTACGGAAAGC |  |  |
| ESGS124 | G-SSR | F: GCGAGGTAAAAAGTATAG | 51 | [1] |
|  |  | R: TCACGTTTGAAGCAAGTA |  |  |
| ES-82 | EST-SSR | F: ATTGACAAGCTCCTTGCAGAGAT | 61 | [2] |
|  |  | R: TTTTTCTTCTTGGATTTCTTCCC |  |  |
| ES-75 | EST-SSR | F: ACCACCCTGGAGTAGCCATT | 58.5 | [2] |
|  |  | R: CTGGTCCATCGTCGACTTC |  |  |
| ES-7 | EST-SSR | F: CCTCCTCCGTTACCATGTTG | 61 | [2] |
|  |  | R: CCCTGCTTTTCCCTCTCTG |  |  |
| ES-51 | EST-SSR | F: GAGCTGAGCTGAGAAGAAAACAG | 55.5 | [2] |
|  |  | R: CACAATCATCTCATCTTCCTTCC |  |  |
| ES-352 | EST-SSR | F: CGTCTTCCGCTTCATCTTCTT | 64 | [2] |
|  |  | R: CAAAGATCCAGATCACACCAAAC |  |  |
| ES-322 | EST-SSR | F: GGGTGTGATTCATAAAACGAATG | 55.5 | [2] |
|  |  | R: TCTTTCTCGTGACTGTTCCTTTC |  |  |
| ES-261 | EST-SSR | F: GCTGTTCTCATTGCTGTTGGTAT | 57 | [2] |
|  |  | R: ATTCACGTCAGTTGTTGGAGACT |  |  |
| ES-180 | EST-SSR | F: TAGAGCAACTCTGGCATATCCTT | 62 | [2] |
|  |  | R: TGCTCCAATCAAACAATTAATCA |  |  |
| ES-179 | EST-SSR | F: GCATGTCCTTTCCAAGAACTATAAA | 62 | [2] |
|  |  | R: TGCTCCAATCAAACAATTAATCA |  |  |
| ES-176 | EST-SSR | F: GTATTGGTCTCCTTAGCCTGGTC | 65 | [2] |
|  |  | R: ATGATTCCCAGGACAAAACTGAT |  |  |
| ES-123 | EST-SSR | F: AGCATGAAGCTCGACTGTGAGT | 55.5 | [2] |
|  |  | R: GCGAGTACATCTCGTACTTCTGG |  |  |
| ES-105 | EST-SSR | F: GGTGGAGAAGGGAGATGAGTC | 61.5 | [2] |
|  |  | R: AGGCTCATGAGGAACAAGTCTCT |  |  |
| Elymus 5264 | EST-SSR | F: ATTGTTTGGGTACCGGTTTATGC | 59 | [3] |
|  |  | R: GCCTGGGTACAGCTGAAACATTAG |  |  |
| Elymus 3592 | EST-SSR | F: TGTTGACAAAAGCAGTTGAAGGG | 61 | [3] |
|  |  | R: GATTTGACCATGGACTGCTTCAC |  |  |
| Elymus 3207 | EST-SSR | F: GTACAAGATCGCCAACTCGAAGAT | 61 | [3] |
|  |  | R: CCTACAGGAAAAGGACTCGTGACT |  |  |
| Elymus 2644 | EST-SSR | F: GAATGGAGCTCCGCTTTAAGATTT | 59 | [3] |
|  |  | R: GCTCCAAACACGGTATAACTCCAC |  |  |
| Cn-48 | EST-SSR | F: TTCTTCAAGGCTATTGTTGCTTC | 61.5 | [4] |
|  |  | R: CGCCTCCTGATTCTCTTCCT |  |  |
| Cn-479 | EST-SSR | F: GTTCGACCACACGCTCAAC | 64 | [4] |
|  |  | R: GCGCTGGCTCACATATACTTCTA |  |  |
| Cn-362 | EST-SSR | F: TGAAATTCTAGCTTCTTCTCCCC | 63 | [4] |
|  |  | R: TAATCTTGCAAGCAGATCCAAAC |  |  |
| Cn-350 | EST-SSR | F: CAGAGTGGGTTCACAGTCCATAG | 64 | [4] |
|  |  | R: CAGCAACAGCAACAACTACAGG |  |  |
| Cn-306 | EST-SSR | F: ACTTCTTCATCTCCAATGCCTTT | 64 | [4] |
|  |  | R: CAGACGAACCAGAGACAGCAT |  |  |
| Cn-299 | EST-SSR | F: CCCCTCTGCTTTCTTGTTATCTT | 58.5 | [4] |
|  |  | R: GTCGTTGAGATCCCACATGC |  |  |
| Cn-294 | EST-SSR | F: ATCATCGTCATCGTCCTCAAC | 63 | [4] |
|  |  | R: AACGTGTTCGCGGCTCTC |  |  |
| Cn-291 | EST-SSR | F: GGCTCAAAAGGTTGAACAGACTA | 57 | [4] |
|  |  | R: CTTCTTGTTTCTCCTCTTCTGGG |  |  |
| Cn-278 | EST-SSR | F: CATGCAAACACTACCTAGAGGGT | 65 | [4] |
|  |  | R: TCATCACTTCTTGCTTCTTTGGT |  |  |
| Cn-237 | EST-SSR | F: GTCCTGTTAGCCAATATGCATTC | 61 | [4] |
|  |  | R: ATTTCTTCCACAAGCAACAACTC |  |  |
| Cn-227 | EST-SSR | F: ATCCCATTCAAGACCAAGTCCTA | 59 | [4] |
|  |  | R: GTAACACGATTTGATGGGAAGAG |  |  |
| Cn-204 | EST-SSR | F: ATCCTCTTCCTGTATTCCTTTGC | 66 | [4] |
|  |  | R: AACCACTGACTCTCGTCCACTC |  |  |
| Cn-193 | EST-SSR | F: CTGCCACTTTTGACCTGTCTC | 57 | [4] |
|  |  | R: GCCTTTCTGTCTCCTGTACCATA |  |  |
| Cn-159 | EST-SSR | F: AGGAGTAGTAGCTGCTCCCCAG | 64 | [4] |
|  |  | R: AATGCTGGATGGGGTGGT |  |  |
