# Supplementary material for: Revelation of genetic diversity and structure of wild Elymus excelsus (Poaceae: Triticeae) collection from western China by SSR markers
Source: PeerJ. 2019 Nov 12;7:e8038. doi: 10.7717/peerj.8038 (PMC6857585; doi:10.7717/peerj.8038)
Supplement: Table S3 [file peerj-07-8038-s005.docx]

|  | Ee 01 | Ee 02 | Ee 03 | Ee 04 | Ee 05 | Ee 06 | Ee 07 | Ee 18 | Ee 08 | Ee 09 | Ee 19 | Ee 17 | Ee 20 | Ee 14 | Ee 10 | Ee 21 | Ee 22 | Ee 23 | Ee 11 | Ee 15 | Ee 12 | Ee 16 | Ee 24 | Ee 25 | Ee 13 |
| --- | --- | --- | --- | --- | --- | --- | --- | --- | --- | --- | --- | --- | --- | --- | --- | --- | --- | --- | --- | --- | --- | --- | --- | --- | --- |
| Ee 01 | 1.00 |  |  |  |  |  |  |  |  |  |  |  |  |  |  |  |  |  |  |  |  |  |  |  |  |
| Ee 02 | 0.93 | 1.00 |  |  |  |  |  |  |  |  |  |  |  |  |  |  |  |  |  |  |  |  |  |  |  |
| Ee 03 | 0.92 | 0.92 | 1.00 |  |  |  |  |  |  |  |  |  |  |  |  |  |  |  |  |  |  |  |  |  |  |
| Ee 04 | 0.90 | 0.91 | 0.91 | 1.00 |  |  |  |  |  |  |  |  |  |  |  |  |  |  |  |  |  |  |  |  |  |
| Ee 05 | 0.90 | 0.90 | 0.93 | 0.95 | 1.00 |  |  |  |  |  |  |  |  |  |  |  |  |  |  |  |  |  |  |  |  |
| Ee 06 | 0.92 | 0.88 | 0.91 | 0.89 | 0.89 | 1.00 |  |  |  |  |  |  |  |  |  |  |  |  |  |  |  |  |  |  |  |
| Ee 07 | 0.90 | 0.90 | 0.91 | 0.88 | 0.89 | 0.91 | 1.00 |  |  |  |  |  |  |  |  |  |  |  |  |  |  |  |  |  |  |
| Ee 18 | 0.91 | 0.87 | 0.90 | 0.86 | 0.88 | 0.89 | 0.87 | 1.00 |  |  |  |  |  |  |  |  |  |  |  |  |  |  |  |  |  |
| Ee 08 | 0.90 | 0.87 | 0.86 | 0.89 | 0.87 | 0.87 | 0.83 | 0.84 | 1.00 |  |  |  |  |  |  |  |  |  |  |  |  |  |  |  |  |
| Ee 09 | 0.90 | 0.91 | 0.89 | 0.91 | 0.90 | 0.88 | 0.87 | 0.88 | 0.89 | 1.00 |  |  |  |  |  |  |  |  |  |  |  |  |  |  |  |
| Ee 19 | 0.89 | 0.86 | 0.85 | 0.89 | 0.88 | 0.89 | 0.86 | 0.86 | 0.88 | 0.93 | 1.00 |  |  |  |  |  |  |  |  |  |  |  |  |  |  |
| Ee 17 | 0.86 | 0.87 | 0.87 | 0.88 | 0.87 | 0.85 | 0.83 | 0.85 | 0.87 | 0.91 | 0.89 | 1.00 |  |  |  |  |  |  |  |  |  |  |  |  |  |
| Ee 20 | 0.86 | 0.84 | 0.85 | 0.86 | 0.86 | 0.85 | 0.82 | 0.84 | 0.85 | 0.88 | 0.87 | 0.84 | 1.00 |  |  |  |  |  |  |  |  |  |  |  |  |
| Ee 14 | 0.90 | 0.88 | 0.87 | 0.88 | 0.87 | 0.89 | 0.86 | 0.88 | 0.86 | 0.89 | 0.89 | 0.85 | 0.91 | 1.00 |  |  |  |  |  |  |  |  |  |  |  |
| Ee 10 | 0.87 | 0.87 | 0.87 | 0.89 | 0.87 | 0.85 | 0.85 | 0.82 | 0.85 | 0.89 | 0.87 | 0.84 | 0.84 | 0.83 | 1.00 |  |  |  |  |  |  |  |  |  |  |
| Ee 21 | 0.87 | 0.84 | 0.85 | 0.87 | 0.84 | 0.84 | 0.82 | 0.85 | 0.84 | 0.89 | 0.89 | 0.87 | 0.86 | 0.85 | 0.83 | 1.00 |  |  |  |  |  |  |  |  |  |
| Ee 22 | 0.85 | 0.81 | 0.83 | 0.84 | 0.83 | 0.85 | 0.81 | 0.85 | 0.83 | 0.87 | 0.89 | 0.87 | 0.86 | 0.86 | 0.83 | 0.90 | 1.00 |  |  |  |  |  |  |  |  |
| Ee 23 | 0.87 | 0.84 | 0.84 | 0.86 | 0.86 | 0.84 | 0.83 | 0.85 | 0.84 | 0.88 | 0.88 | 0.87 | 0.87 | 0.87 | 0.84 | 0.89 | 0.92 | 1.00 |  |  |  |  |  |  |  |
| Ee 11 | 0.86 | 0.87 | 0.86 | 0.88 | 0.86 | 0.85 | 0.83 | 0.79 | 0.84 | 0.87 | 0.89 | 0.87 | 0.82 | 0.83 | 0.85 | 0.84 | 0.84 | 0.85 | 1.00 |  |  |  |  |  |  |
| Ee 15 | 0.83 | 0.79 | 0.82 | 0.83 | 0.81 | 0.80 | 0.79 | 0.82 | 0.82 | 0.85 | 0.85 | 0.84 | 0.81 | 0.83 | 0.80 | 0.83 | 0.84 | 0.83 | 0.81 | 1.00 |  |  |  |  |  |
| Ee 12 | 0.84 | 0.83 | 0.85 | 0.85 | 0.83 | 0.81 | 0.81 | 0.82 | 0.78 | 0.82 | 0.81 | 0.80 | 0.77 | 0.81 | 0.79 | 0.81 | 0.79 | 0.82 | 0.82 | 0.80 | 1.00 |  |  |  |  |
| Ee 16 | 0.81 | 0.78 | 0.80 | 0.82 | 0.81 | 0.83 | 0.80 | 0.80 | 0.79 | 0.81 | 0.84 | 0.80 | 0.83 | 0.83 | 0.77 | 0.82 | 0.83 | 0.82 | 0.78 | 0.80 | 0.76 | 1.00 |  |  |  |
| Ee 24 | 0.81 | 0.77 | 0.79 | 0.79 | 0.78 | 0.80 | 0.79 | 0.78 | 0.74 | 0.78 | 0.77 | 0.77 | 0.76 | 0.79 | 0.73 | 0.80 | 0.78 | 0.81 | 0.76 | 0.76 | 0.78 | 0.79 | 1.00 |  |  |
| Ee 25 | 0.81 | 0.79 | 0.80 | 0.78 | 0.79 | 0.82 | 0.82 | 0.79 | 0.75 | 0.79 | 0.77 | 0.76 | 0.79 | 0.82 | 0.74 | 0.78 | 0.76 | 0.78 | 0.73 | 0.74 | 0.75 | 0.81 | 0.88 | 1.00 |  |
| Ee 13 | 0.78 | 0.78 | 0.76 | 0.79 | 0.77 | 0.79 | 0.75 | 0.73 | 0.73 | 0.76 | 0.77 | 0.76 | 0.72 | 0.76 | 0.76 | 0.73 | 0.72 | 0.74 | 0.77 | 0.68 | 0.75 | 0.67 | 0.70 | 0.67 | 1.00 |
