# Supplementary material for: Revelation of genetic diversity and structure of wild Elymus excelsus (Poaceae: Triticeae) collection from western China by SSR markers
Source: PeerJ. 2019 Nov 12;7:e8038. doi: 10.7717/peerj.8038 (PMC6857585; doi:10.7717/peerj.8038)
Supplement: Table S4 [file peerj-07-8038-s006.docx]

|  | XJC | SCC | GSC |
| --- | --- | --- | --- |
| XJC | - |  |  |
| SCC | 0.084** | - |  |
| GSC | 0.297** | 0.012* | - |
